# A Neurotoxic Snake Venom without Phospholipase A_2_: Proteomics and Cross-Neutralization of the Venom from Senegalese Cobra, *Naja senegalensis* (Subgenus: *Uraeus*)

**DOI:** 10.3390/toxins13010060

**Published:** 2021-01-14

**Authors:** Kin Ying Wong, Kae Yi Tan, Nget Hong Tan, Choo Hock Tan

**Affiliations:** 1 Department of Pharmacology, Faculty of Medicine, University of Malaya, Kuala Lumpur 50603, Malaysia; kinying12@gmail.com; 2 Department of Molecular Medicine, Faculty of Medicine, University of Malaya, Kuala Lumpur 50603, Malaysia; kytan_kae@um.edu.my (K.Y.T.); tanngethong@yahoo.com.sg (N.H.T.)

**Keywords:** *Naja* (*Uraeus*) *senegalensis*, *Naja haje* complex, venomics, snakebite envenomation, immunoreactivity, antivenom neutralization

## Abstract

The Senegalese cobra, *Naja senegalensis*, is a non-spitting cobra species newly erected from the *Naja haje* complex. *Naja senegalensis* causes neurotoxic envenomation in Western Africa but its venom properties remain underexplored. Applying a protein decomplexation proteomic approach, this study unveiled the unique complexity of the venom composition. Three-finger toxins constituted the major component, accounting for 75.91% of total venom proteins. Of these, cardiotoxin/cytotoxin (~53%) and alpha-neurotoxins (~23%) predominated in the venom proteome. Phospholipase A_2_, however, was not present in the venom, suggesting a unique snake venom phenotype found in this species. The venom, despite the absence of PLA_2_, is highly lethal with an intravenous LD_50_ of 0.39 µg/g in mice, consistent with the high abundance of alpha-neurotoxins (predominating long neurotoxins) in the venom. The hetero-specific VINS African Polyvalent Antivenom (VAPAV) was immunoreactive to the venom, implying conserved protein antigenicity in the venoms of *N. senegalensis* and *N. haje*. Furthermore, VAPAV was able to cross-neutralize the lethal effect of *N. senegalensis* venom but the potency was limited (0.59 mg venom completely neutralized per mL antivenom, or ~82 LD_50_ per ml of antivenom). The efficacy of antivenom should be further improved to optimize the treatment of cobra bite envenomation in Africa.

## 1. Introduction

Each year, snakebite envenomation causes a death toll that surpasses 100,000, and approximately three times as many permanent disabilities and psychological complications in those survived [1]. Most of the victims are from impoverished and remote populations, typically those engaging in agricultural activities [2]. The exact morbidity and mortality of snakebite envenomation are, however, greatly underestimated due to the scarcity of reliable epidemiological data worldwide, in particular rural areas where health systems are suboptimal, and people have limited access to proper treatment. As such, in 2017 snakebite envenomation was reinstated as a priority neglected tropical disease by the World Health Organization [3]. To solve this long persistent global health crisis, key strategies were proposed to combat the various challenges faced [4]. One of the “pillar strategies” proposed is the provision of effective and safe treatment that entails the use of appropriate antivenom, for which its species-specific efficacy needs to be addressed.

Unfortunately, venomous snakes are highly diverse, with at least 200 species being medically important and widely distributed in different geographical areas [5]. In Africa and Asia, the cobras (genus: *Naja*) are probably the most commonly encountered and medically important venomous snakes. Systemic neurotoxicity and local tissue necrosis are the hallmark features of cobra envenomation, while the severity differs among species. There are approximately 25 extant cobra species (*Naja* spp.), and the venom composition of each species vary considerably in terms of the relative abundances and subtypes of toxins, although the three-finger toxins and phospholipases A_2_ are generally conserved [6,7,8,9]. Variations in the venom composition give rise to differential toxicity and clinically variable neutralization response to antivenom treatment. Thus, it is critical to understand the composition profile and toxicity neutralization of cobra venom according to the species and geographical origin, so that the pathophysiology of envenomation can be elucidated and treatment can be improved.

**Figure 1:**
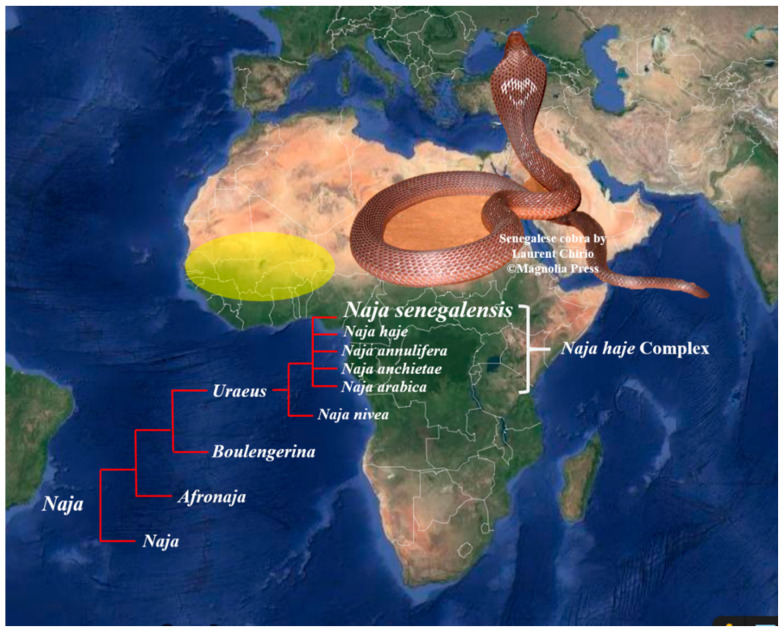
Geographical distribution of *Naja senegalensis* in Western Africa, encompassing Senegal, Gambia, Mali, Burkino Faso, Ghana, Benin, Guinea-Bissau, Nigeria, and the Ivory Coast (yellow region) [5]. Insets: *Naja senegalensis* from the subgenus of *Uraeus*, elevated as a new species from the *Naja haje* complex. Note the distinct hood marking which is uncommon in African but Asiatic cobras. Snake photograph was courtesy of Laurent Chirio with copyright © Magnelia Press 2009 [12].

The present systematics places the diverse cobra species under four subgenera, i.e., *Naja*, *Afronaja*, *Boulengerina,* and *Uraeus*, with the latter three being African cobras. In the past two decades, the systematics of non-spitting African cobras, historically clustered under the *Naja haje* complex, has been extensively revised based on molecular markers (mitochondria DNA) and morphological characters, resulting in the elevation of several distinct species from within the complex. These relatively new species include *Naja annulifera* [10], *Naja anchietae* [11], *Naja arabica,* and *N. senegalensis* [12], which are assigned to the subgenus of *Uraeus* [13] along with *Naja haje* and *Naja nivea*. Among the six *Uraeus* cobra species, *N. senegalensis* (Senegalese cobra) is an endemic species distributed in Western Africa across Senegal, Gambia, Mali, Burkino Faso, Ghana, Benin, Guinea-Bissau, Nigeria, and the Ivory Coast [14,15] (Figure 1). The distinctive morphological characteristics (with a high number of scale rows around the neck) and genetic differences (unique mtDNA haplotypes) of *N. senegalensis* separated it from the other members of *N. haje* complex. An adult *N. senegalensis* may possess a considerably huge body size with a length up to 2.3 m. Adult snakes are dark-grayish brown dorsally and yellowish ventrally, with a dark collar around the neck, and occasionally bearing a heart-shaped nuchal mark on the hood (Figure 1) [12]. Clinically, envenomation by *N. senegalensis* has been reported to cause prominent neuromuscular paralysis with minimal local tissue damage [16], but little is known about the composition, pathophysiology, and neutralization of the venom of this new cobra species. Nonetheless, a recent study showed that the *N. senegalensis* venom was void of PLA_2_ activity [17], suggesting a venom phenotype that is unique to the species and perhaps the entire monophyletic clade of the *Uraeus* subgenus. This was further supported by the venomics of another non-spitting African cobra, *N. annulifera* (sub-Saharan snouted cobra), whose venom proteome is indeed deficient of PLA_2_ [18]. The presumed “loss” of PLA_2_ in *N. senegalensis* venom, and possible altered venom toxicity resulted therefrom, remain to be further validated. Hence, we sought to investigate the venom composition of *N. senegalensis* applying a protein decomplexing strategy of venomics, where chromatographic fractions of the venom were analyzed with nano-liquid chromatography-tandem mass spectrometry (nano-LCMS/MS) for protein identification and quantification [8]. Furthermore, the lethality of the venom, and the immunological binding activity, as well as the cross-neutralization efficacy of a hetero-specific antivenom marketed in Africa (VINS African Polyvalent Antivenom), were examined against *Naja senegalensis* venom. The findings provided insights into the pathophysiology and management of *N. senegalensis* envenomation in Western Africa.

## 2. Results and Discussion

### 2.1. Chromatographic Separation of Naja Senegalensis Venom

**Figure 2:**
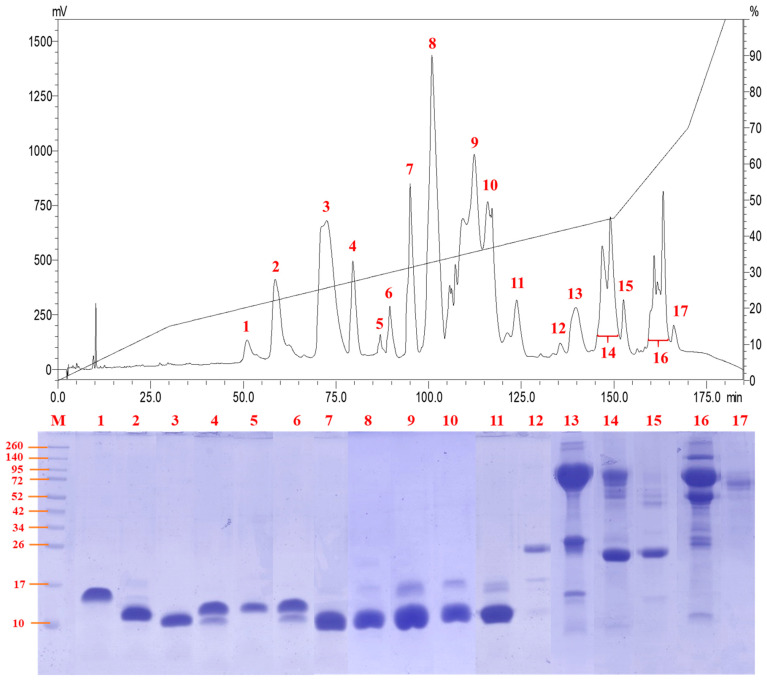
Reverse-phase high-performance liquid chromatography of *Naja senegalensis* venom. Chromatogram of the venom fractionation (top panel) and separation of venom fractions using SDS-PAGE under reducing condition (bottom panel). M, protein ladder for estimating the molecular weight of sample proteins.

C18 reverse-phase HPLC resolved the venom proteins into 17 fractions of increasing hydrophobicity (Figure 2). SDS-PAGE of the fractions showed that the majority of the venom proteins were of low molecular weight (<20 kDa) under reducing conditions (Figure 2), although some proteins might exist as complexes in their native forms. Most of the low molecular weight proteins (fractions 1 to 11) were eluted between 50 and 125 min of RP-HPLC, and these made up approximately 80% of the total venom proteins. Proteins with middle (25–35 kDa) to high molecular weights (>40 kDa) were eluted through fractions 12 to 17 between 135 and 170 min of HPLC, together accounting for <20% of total venom proteins. The predominant presence of low molecular weight proteins in *N. senegalensis* venom is a typical venom phenotype of cobras irrespective of phylogeographical distribution, as observed in a number of Asiatic and African cobra venoms including Indian *N. naja* [19], Indonesian *N. sputatrix* [9], Philippine *N. philippinensis* [8], Thai *N. kaouthia* [20], and African *N. annulifera* [18] whose venoms were profiled under the same experimental conditions.

### 2.2. Venom Proteomes and Toxicity Correlation

**Figure 3:**
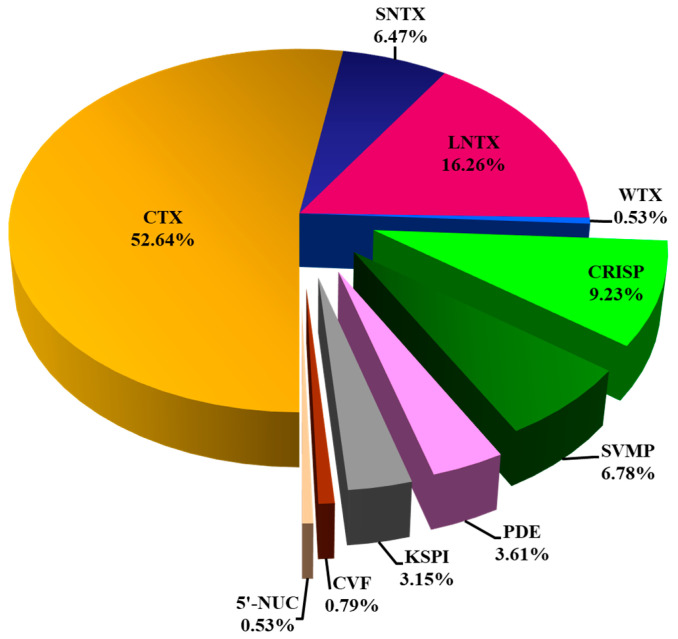
Snake venom proteome of *Naja senegalensis*. Proteomic profiling was accomplished with QTOF nano-ESI-LCMS/MS of reverse-phase HPLC-fractionated venom proteins. All protein samples were subjected to in-solution tryptic digestion. Abbreviations: CTX, cytotoxin/cardiotoxin; SNTX, short neurotoxin; LNTX, long neurotoxin; WTX, weak neurotoxin; CRISP, cysteine-rich secretory protein; CVF, cobra venom factor; KSPI, Kunitz-type serine protease inhibitor; PDE, phosphodiesterase; SVMP, snake venom metalloproteinase; 5′-NUC, 5′-nucleotidase. CTX, SNTX, LNTX, and WTX constituted three-finger toxins (3FTX) superfamily.

Nano-ESI-LCMS/MS analysis of the *N. senegalensis* venom fractions with QTOF mass spectrometry identified a total of 54 proteins (Table 1). Of these, 37 proteins were non-redundant distinct proteoforms categorized into 7 protein families (Figure 3). Details of protein identification from both mass spectrometry analyzers (matched sequences, peptide scores, mass-to-charge ratios, annotated accession codes, and corresponding species) were provided in Supplementary Files S1. All proteins identified were annotated to homologous proteins from closely related elapid species but not *N. senegalensis*, for there is a total lack of toxin database specific to this newly erected species. Regardless, the three-finger toxins (3FTX) superfamily constituted the most dominantly expressed proteins (with relative abundance >75%) in the venom proteome of *N. senegalensis*. Of note, the 3FTX were also the low molecular weight proteins eluted from the RP-HPLC of the venom (Figure 2), and within this protein family, the cytotoxins (also named as cardiotoxins, CTX) were predominated. Cysteine-rich secretory proteins (CRISP) were the second most abundant proteins in the venom, constituting about 9% of the total venom proteins. The remaining minor protein components in the venom included snake venom metalloproteinase protein (SVMP), phosphodiesterase (PDE), Kunitz-type serine protease inhibitor (KSPI), cobra venom factor (CVF), and 5′-nucleotidase (5′-NUC), altogether accounting for <20% of the total venom proteins.

Three-finger toxins (3FTX) of *N. senegalensis* venom were formed by the subfamilies/subgroups of short and long neurotoxins, weak neurotoxins, and cytotoxins. Of these, the cytotoxins or cardiotoxins (CTX) were the most diversely and abundantly expressed, comprising 14 distinct CTX proteoforms at 53% of the total venom proteins (Table 2). CTXs are commonly found in moderate to high abundances in cobra venoms [6,19]. Genomics and venom-gland transcriptomics of Asiatic cobras revealed that CTX multigenes are diversely and abundantly expressed while retaining high structural homology of the proteins [21,22,23]. The amount of CTX in the venom, however, can vary considerably amongst various cobra species, ranging from 20% to 86% of total venom proteins [6,7,8,9,19,24,25]. The vast dynamic range of CTX in a cobra venom presumably reflects the phenotypic adaptation of distinct species to a different ecological niche, although CTX generally serves a common function related to the cytotoxic and tissue-damaging effects. Cobras typically employ alpha-neurotoxins to paralyze prey (primary hunting strategy); hence, the abundantly expressed CTXs are likely needed for digestive and defensive (algesic) purposes [26,27]. In clinical envenomation, CTX is implicated in local tissue necrosis and venom ophthalmia [28,29], but it is unlikely the direct lethal factor of the venom since its lethal effect is much weaker compared to alpha-neurotoxins (intravenous median lethal dose, LD_50_ of CTX > 1.0 µg/g, c.f. LD_50_ of alpha-NTX < 0.2 µg/g, tested in mice) [30,31]. The cytotoxic activities of CTX are, nevertheless, pharmacologically diverse. Depending on the dose of CTX, the cell death could be caused by apoptosis or necrosis [32] mediated through various mechanisms, e.g., mitochondrial fragmentation (CTX binding to cardiolipin) and membrane pore formation (CTX interacting electrostatically with anionic lipids) [33,34]. Earlier studies also showed that some cobra CTXs could elicit cardiotoxicity through disrupting calcium regulation in cardiac muscle cells, thus prolonging membrane depolarization, resulting in systolic heart arrest [35,36]. Cardiotoxicity, however, has not been well recognized in clinical envenomation and death caused by most cobra bites, where respiratory failure following systemic paralysis is the main cause of death. The tissue-damaging effect of cobra CTX, however, is variable across different species, with cytotoxic potential that appears to rise with the hood-displaying behavior and aposematic coloration to warn off predators or aggressors [27]. The high abundance of CTX in *N. senegalensis* venom suggests its important role in the pathophysiology of envenomation, at least in tissue-damaging activities. Unfortunately, clinical data of envenomation caused by *N. senegalensis* remains scarce to allow further interpretation of the medical relevance of its CTX.

The alpha-neurotoxins (SNTX and LNTX) constituted the second major group of 3FTX proteins in the *N. senegalensis* venom (Table 2). These are post-synaptic neurotoxins that bind and block nicotinic acetylcholine receptors at the neuromuscular junction, leading to systemic paralysis, respiratory failure, and death [37]. A clear correlation exists between the abundance of alpha-neurotoxins and the lethal potency of cobra venoms [8], and the high abundance of alpha-neurotoxins in *N. senegalensis* venom (up to ~23% of total venom proteins) indeed correlated well with its neurotoxic activity and potent lethality (*i.v.* LD_50_ = 0.39 µg/g), as observed in this study (Table 3). The finding is also consistent with the prominent neurotoxic effect reported in clinical envenomation caused by the Senegalese cobra [16]. Furthermore, the quantitative composition of alpha-neurotoxins in *N. senegalensis* venom (current study) appeared to be higher than that reported in other African cobra species (α-neurotoxins < 20% of total venom proteins), whose venom LD_50_ were correspondingly higher (0.53–1.42 µg/g) [6,18,25,38], supporting that alpha-NTX is the principal toxin that drives the lethality of the cobra venom. In brief, alpha-neurotoxins consist of Type I (short neurotoxins, SNTX) and Type II (long neurotoxins, LNTX), both of which are highly neurotoxic with LD_50_ values well established between 0.05–0.2 µg/g for cobras [8,9,30,31,39,40]. The SNTX and LNTX, however, differ in the length of the amino acid sequence, the number of disulfide bonds, the type of binding receptor, and affinity [41,42]. We previously reported that in cobra venoms, the relative abundance ratio of LNTX to SNTX can be strikingly variable among different cobra venoms. The subproteomic variation in LNTX and SNTX composition has an impact on the toxicity of venom and its neutralization by antivenom [30,31]. In the present work, the abundance of long neurotoxins (LNTX) in *N. senegalensis* venom was much higher than that of short neurotoxins (SNTX), implying that the neurotoxic activity (and hence the lethality) of the venom is mediated primarily by LNTX. The similar phenotypic predilection for a LNTX-predominating venom is observed in most neurotoxic Asiatic cobras, including the Thai *N. kaouthia* (LNTX:SNTX ~ 4:1) [20], Thai *N. siamensis* (LNTX:SNTX ~ 4:1) [7], Malaysian *N. sumatrana* (LNTX:SNTX ~ 4:1) [43], and Pakistani *N. naja* venom (LNTX:SNTX ~ 3–5:1) [19,44]. On the other hand, SNTX-predominating cobra venoms were found mainly in species inhabiting the eastern part of Asia, including the Taiwanese *N. atra* (SNTX:LNTX ~ 10:1) [45], Javan *N. sputatrix* (SNTX: LNTX ~ 16:1) [9], and the Philippine *N. philippinensis* which represents the most exclusive case of extreme bias to SNTX expression (SNTX:LNTX ~ 45:1) [8]. Although SNTX and LNTX are essentially both post-synaptic acting neurotoxins, recent studies indicated that these toxins are not necessarily equipotent, as SNTX may produce a more reversible blockage of nicotinic acetylcholine receptor (nAChR) compared to LNTX [46,47,48]. The present proteomic finding thus supports that the antivenom production for use in Western Africa should be tailored to reverse the toxicity of the alpha-neurotoxins, in particular the LNTX in *N. senegalensis* venom.

In addition to CTX and α-NTX, the *N. senegalensis* venom also contained minute weak neurotoxins (WTX, 0.5% of total venom proteins). WTX interacts with muscle and neuronal nAChR at low affinity, and has toxicity that is 50 to 100 times lower than α-NTX (WTX LD_50_ = 5–80 µg/g c.f. α-NTX LD50 = 0.05–0.2 µg/g) [49,50]. As such, these toxins which were minimally expressed in the proteome, likely do not play a major role in the pathophysiology of envenomation, although their biological significance in the evolution of cobra venom remains to be elucidated.

### 2.3. Other Protein Constituents

Other components present in the *N. senegalensis* venom proteome were proteins with higher molecular weights (>25 kDa). Of these, cysteine-rich secretory protein (CRISP) shows a broad range of biological activities, e.g., increasing vascular permeability and promoting inflammatory response which may be implicated in the pathogenesis of envenomation [51,52,53]. Snake venom metalloproteinase (SVMP) are multi-domain, zinc-dependent enzymatic toxins abundantly present in viperid and crotalid venoms, and contribute to hemotoxic envenomation [54,55]. SVMPs, typically the P-III subtype, have been discovered from most cobra venoms (as in the current study) but the amount is usually very low and the function is unlikely related to hemotoxicity. A Kunitz-type serine protease inhibitor was also detected in the current study. KSPIs contribute to neurotoxic activities of krait and mamba venoms [56,57,58]; however, they are not known to implicate in neurotoxic envenomation caused by cobras.

The presence of phosphodiesterase (PDE), 5′-nucleotidase (5′-NUC), and cobra venom factor (CVF) in the *N. senegalensis* venom suggested their involvement in facilitating the spread of venom toxins. The concomitant hydrolysis of ATP and ADP by PDE and 5′-NUC, respectively, generates adenosine, a potent vasodilator that increases local vascular permeability and thus toxin diffusion further from the bite site [59]. CVF, a complement-activating protein in cobra venom, is structurally and functionally highly homologous to complement component C3. Its presence, although minute in the venom, is believed to also facilitate the distribution of venom toxins via vasodilation [60].

### 2.4. The Lack of Phospholipase A_2_ (PLA_2_) in Naja Senegalensis Venom

Recent proteomic and enzymatic studies demonstrated that amongst cobras, species of the *Uraeus* subgenus appeared to have venoms that lack secretory PLA_2_ [17,18]. The phenomenon is, again, revealed in the present work tested on *N. senegalensis* venom. We fractionated the venom through C18 RP-HPLC as per the decomplexing venomic protocol [61], but the subsequent QTOF tandem mass spectrometry analysis did not detect any PLA_2_ which was expected to co-elute, typically, with the hydrophobic CTX [8,9,19]. The finding is consistent with the negligible PLA_2_ enzymatic activity in the venoms of *N. senegalensis* and representative species of the *Uraeus* subgenus (*N. haje*, *N. annulifera,* and *N. nivea*), contrary to the other Asiatic and African cobra species (*Naja*, *Afronaja* and *Boulengerina* subgenera) which have evolved venom phenotype characterized by abundantly expressed PLA_2_ [17]. It has been shown that positive selection has a dominant role on elapid (cobra) PLA_2_, in which the PLA_2_ multigene evolved from an ancestral non-toxic PLA_2_ through repeated gene duplication, followed by functional divergence [62]. The resultant increases in genomic (multigene) and phenotypic (venom composition) complexity are deemed essential to allow the cobras to adapt to ecological niche shifts and new prey types. Our present finding, however, suggests that the PLA_2_ gene in at least one cobra species underwent purifying selection instead. Based on the observed phenomenon, we speculate a possible scenario where post-speciation niche shifts subjected the PLA_2_ multigene to the birth-and-death and “selective sieve” processes of gene duplication, divergence, and loss, in a way similar to the evolution of the elapid three-finger toxins as reported previously [63,64]. Depending on the functionality of the PLA_2_, genes that are no longer effective in subduing new prey species or deterring predators were lost as in pseudogenization—a more common fate for a duplicated gene actually. Collectively, the event contributes to shaping the diversity of the venom in its present form. The observation warrants further genetic analysis, and we anticipate genomic and venom-gland transcriptomic studies to further shed light on the evolution of snake venom PLA_2_ in *N. senegalensis*, and related cobra species from the *Uraeus* subgenus.

### 2.5. Immunoreactivity and Neutralization of Antivenom

**Figure 4:**
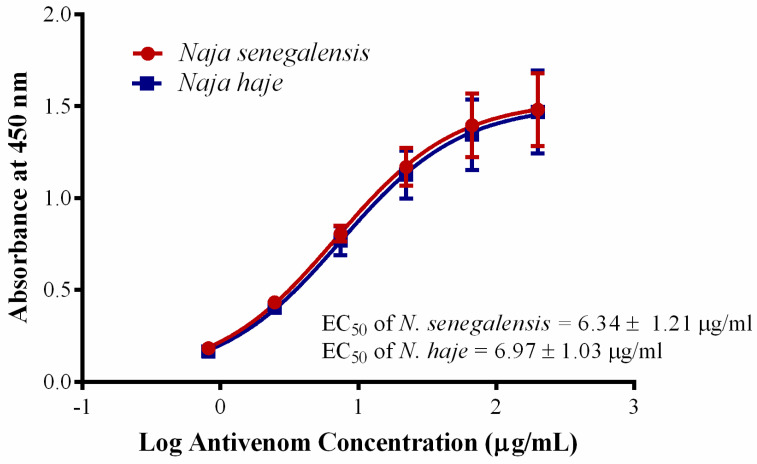
Immunological binding activity of VINS African Polyvalent Antivenom (VAPAV) toward *N. senegalensis* and *N. haje* venoms. The immunological binding activity was represented as half the maximum concentration (EC_50_). Values were means ± S.E.M. of triplicates.

Cobras are common causes of snakebite envenomation in Sub-Saharan Africa, causing two distinctive toxic manifestations. The African non-spitting cobras (*N. haje*, *N. nivea*, *N. senegalensis*, *N. annulifera*) and the forest cobra (*N. melanoleuca*) cause systemic neurotoxic envenomation, while the African spitting cobras (*N. nigricollis*, *N. katiensis*, *N. mossambica*, *N. pallida*, *N. ashei*) cause predominantly local cytotoxic envenomation (tissue necrosis) [65]. Due to the limited local capacity for antivenom production in Africa, currently, various antivenom products are imported and marketed in Sub-Saharan Africa; these include SAIMR polyvalent antivenom produce from South Africa, Antivipmyn-Africa from Mexico, VACSERA from Egypt, EchiTAb-Plus-ICP from Costa Rica, VINS Pan Africa antivenom and ASNA antivenom from India, and Inoserp Pan Africa from Mexico with limited information regarding their clinical effectiveness [66]. Considering that *N. senegalensis* is a highly neurotoxic species (venom LD_50_ = 0.39 µg/g, current study) and widely distributed in Western Africa, an effective antivenom is undoubtedly the definitive treatment needed to treat the envenomation caused by this cobra. Unfortunately, up to now, there is still no species-specific antivenom available to treat its envenomation. Hetero-specific polyvalent antivenom products, e.g., VAPAV are available for envenomation caused by *N. haje* but the cross-neutralization efficacy against *N. senegalensis* venom has not been examined. In this study, VAPAV showed dose-dependent immunological cross-reactivity toward *N. senegalensis* venom (Figure 4), with immunological binding activity (indicated by half-maximal effective concentration, EC_50_ = 6.34 ± 1.21 µg/mL) that was comparable to its binding to the homologous *N. haje* venom (EC_50_ = 6.97 ± 1.03 µg/mL) (*p* > 0.05). The finding revealed conserved protein antigenicity in the venoms of *N. senegalensis* and *N. haje*, which are phylogenetically related species within the *Uraeus* genus. The cross-reactivity suggested cross-neutralization capability of VAPAV, which we subsequently evaluated with the WHO-recommended antivenom efficacy test (in vivo neutralization test) [67,68]. Our finding showed that the hetero-specific VAPAV was able to cross-neutralize the lethality of Senegalese cobra venom moderately, with a median effective dose (ED_50_) of 60.81 µL against 5 × LD_50_ challenge dose of the venom. In terms of neutralizing potency, each milliliter of VAPAV was able to completely neutralize 0.59 mg of the venom. The protein concentration of VAPAV was 130.68 mg/mL (by bicinchoninic acid assay).

In this study, the neutralization of VAPAV against the Senegalese cobra venom was moderately effective. Its neutralization potency was determined as 0.59 mg/mL, which falls within the range of potency reported for a few African antivenom products tested in vivo: 0.31–1.20 mg venom neutralized per mL of antivenom, in mice against African cobra venoms [18,66,69,70,71] (Table 3). However, the neutralization potency (mg/mL) has a limitation when compared between different antivenom products against different venoms, as venom toxicity varies among different cobra species. Therefore, the protective efficacy (R, defined as the number of LD_50_ neutralized per mL antivenom at which 50% of mice survived) was included as an indicator for comparative purpose. The protective efficacy (R) of VAPAV against *N. senegalensis* venom was 82.2 LD_50_/_mL_ antivenom, indicating a relatively high efficacy when compared amongst other African antivenoms against the venoms of *Uraeus* species (R = 9.8–112.3 LD_50_/_mL_). In general, the neutralization potency of these commonly used African antivenoms rarely exceeded 1 mg venom/mL antivenom; this poses a challenge in the clinical dosing of antivenom as an adult cobra can easily inject a large quantity of venom beyond 50–100 mg. In the case of *N. senegalensis*, assuming there is an initial systemic bioavailability of 50 mg venom, a starting dose of at least 10 vials of the African antivenom (such as VAPAV) would therefore be required to initiate neutralization. Clinically, the doses often need to be escalated or repeated over time when the residual venom is slowly absorbed from the subcutaneous depot (bite site) while the antivenom is continuously metabolized or eliminated from the body. Furthermore, from the clinical perspective, it is impossible to overlook the cost issue and adverse effects (fatal hypersensitivity reactions) associated with high doses of antivenom. Based on an antivenom costing to Kenyan hospitals, antivenoms produced by non-Africa-based manufacturers (typically from India) were indeed marketed at costs considerably lower than the Africa-based products like SAIMR or Fav Afrique antivenoms—for instance, in 2016 VAPAV was marketed at USD 47.90 per vial *c.f.* SAIMR at USD 315 per vial [71], but the cost of using multiple doses of VAPAV remains prohibitive in the face of extreme poverty in those countries. The finding of the cross-neutralization indicated that the antivenom resources (poly-specific antivenom with an indication for *N. haje* or the Egyptian cobra bite) may be shared in the region for the treatment of envenomation caused by Senegalese cobra, without the need to introduce a new antivenom product into the market. However, the main challenge, i.e., how to increase the potency of the antivenom while ensuring its safety and affordability, remains unresolved.

There are several suggestions to revolutionize antivenom production, e.g., the development of recombinant antivenom [73], development of elapid antivenom by immunizing horses with recombinant consensus short-chain α-neurotoxins [74], and re-formulation of toxin-targeting, pan-species antivenom [75,76]. These new initiatives are, however, challenging in terms of practicality owing to the enormous time and cost needed in R&D, various regulatory constraints, limited market size, and low-profit margin of the product. The problem could be circumvented by repurposing and making use of existing hetero-specific antivenoms which have been shown to confer an effective cross-neutralization effect when a species-specific antivenom is not available [71,72,77]. Besides, the use of small toxin inhibitors such as varespladip, a phospholipase A_2_ inhibitor has been advocated as an adjunct treatment in snakebite envenomation, as it showed a promising effect in attenuating neurotoxicity caused by elapid snake venoms [78,79]. The inhibitor varespladip, however, likely has no use in snakebite envenomation caused by most cobra species whose venom PLA_2_ are not the principal toxins [30,31,80], or, snakes with atypical venoms that contain no PLA_2_, such as the Senegalese cobra (present study). Antivenom thus remains the mainstream and the most realistic treatment of cobra bites, though there is a need to optimize its supply and distribution as well as judicious clinical use in terms of product selection, dosing, and administration.

## 3. Conclusions

This is the first report on the quantitative venom proteomics of *N. senegalensis*, a distinct non-spitting cobra species with medical importance in Western Africa. Cytotoxins/cardiotoxins (CTX) and alpha-neurotoxins from the three-finger toxins family (3FTX) constituted the major proteins of the venom. The long α-neurotoxins were more abundant compared to short α-neurotoxins (LNTX:SNTX = 3:1), suggesting that LNTX plays a more prominent role in the potent neurotoxicity of the venom as reflected by its low LD_50_. The study also uncovered an unusual venom phenotype that is characterized by the distinct lack of PLA_2_, implying that the PLA_2_ multigene in this species and probably the whole lineage of the *Uraeus* subgenus underwent purifying evolution in which they were pseudogenized in adaptation to new dietary shifts and changing ecological niche. The study further revealed well-conserved venom antigenicity in *N. senegalensis* and the phylogenetically related *Naja haje*, as the African polyvalent antivenom, VAPAV was able to immunorecognize the *N. senegalensis* venom and cross-neutralize its in vivo toxicity albeit the potency was low. The findings provide insights into the use of regional antivenom available in Africa, and potentially the improvement of snakebite management in Western Africa where the Senegalese cobra contributes to the disease burden of snakebite envenomation.

## 4. Materials and Methods

### 4.1. Consumables and Reagents

Ammonium bicarbonate, dithiothreitol (DTT), and iodoacetamide (IAA) were purchased from Sigma-Aldrich (St. Louis, MO, USA). MS grade trypsin protease, Spectra™ Multicolor Broad Range Protein Ladder (10 to 260 kDa, catalog number: 26634), and HPLC grade solvents were purchased from Thermo Scientific™ Pierce™ (Rockford, IL, USA). Acetonitrile (HPLC grade), LiChrospher^®^ WP 300 C_18_ RP-HPLC column, and Millipore ZipTip^®^ C_18_ Pipette Tips were obtained from Merck (Kenilworth, NJ, USA). Other chemicals and solvents used were of analytical grade and purchased from Sigma-Aldrich (USA).

### 4.2. Venom and Antivenom

The venoms of *Naja senegalensis* and *Naja haje* (used in immunoreactivity study) were supplied by Latoxan (Valence, France) in lyophilized form and stored at −20 °C until use. The antivenom used was VINS African polyvalent antivenom manufactured by VINS Bioproducts Limited (VAPAV, batch no.: 07AS1604; expired date: February 2020). VAPAV is a purified F(ab’)_2_ obtained from the horse serum hyperimmunized against the venoms of *Naja melanoleuca*, *N. nigricollis*, *N. haje*, *D. polylepis*, *D. viridis*, *D. jamesoni*, *B. gabonica*, *B. arietans*, *E. leucogaster*, and *E. ocellatus*. The antivenom was in lyophilized form and reconstituted in 10 mL of saline water and each mL can neutralize 20 LD_50_ of *N. melanoleuca* and *N. nigricollis* venoms; and 25 LD_50_ of *N. haje*, *D. polylepis*, *D. viridis*, *D. jamesoni*, *B. gabonica*, *B. arietans*, *E. leucogaster*, and *E. ocellatus* venoms. The antivenom was used before the expiration date.

### 4.3. Animals and Ethics Statement

Mice used in this study were of albino ICR strain (20–25 g) supplied by the Animal Experimental Unit from the University of Malaya. The protocol for animal experimentation was carried out based on the Council for International Organizations of Medical Sciences (CIOMS) guidelines [81] and was approved by the Institutional Animal Care and Use Committee of the University of Malaya (Ethics clearance number: 2014-09-11/PHAR/R/TCH, approved 9 November 2014).

### 4.4. Reverse-Phase High-Performance Liquid Chromatography (RP-HPLC)

Three milligrams of *N. senegalensis* venom was reconstituted in 200 µL ultrapure water and the impurities were removed by centrifugation prior subjected to C_18_ reverse-phase high-performance liquid chromatography (HPLC). The LiChrospher^®^ WP 300 C_18_ column (5 µm particle size) column was pre-equilibrated with 0.1% TFA in water (Eluent A) and the sample was eluted with 0.1% TFA in acetonitrile (Eluent B) using a linear gradient of 5% B for 10 min, 5–15% B over 20 min, 15–45% B over 120 min and 45–70% B over 20 min. The flow rate was set at 1 mL/min. Absorbance was monitored at 215 nm and the resulting peaks were collected, lyophilized, and kept at −20 °C prior to use.

### 4.5. Sodium Dodecyl Sulphate-Polyacrylamide Gel Electrophoresis (SDS-PAGE)

The fractions collected from RP-HPLC was reconstituted in ultrapure water and separated by 15% sodium dodecyl sulfate-polyacrylamide gel electrophoresis (SDS-PAGE) under reducing condition at 100 V for 2 h. Thermo Scientific Spectra™ Multicolor Broad Range Protein Ladder (Thermo Scientific™ Pierce™, Rockford, IL, USA) containing 10 prestained proteins ranging from 10 kDa to 260 kDa was used for molecular mass calibration. Gel was stained with Coomassie Brilliant Blue R-250 and the bands were scanned using ImageScanner III (GE Healthcare, Uppsala, Sweden).

### 4.6. Venom Fractions in-Solution (FIS) Tryptic Digestion and Agilent Q-TOF Mass Spectrometry

The protein fractions were reduced with dithiothreitol (DTT), alkylated with iodoacetamide (IAA), and digested with MS grade trypsin (Pierce^TM^) before desalting with C_18_ ZipTip^®^ Pipette Tips. The tryptic peptides were then reconstituted in 7 µL of 0.1% formic acid in water and subjected to nano-electrospray ionization liquid chromatography-tandem mass spectrometry (ESI-LCMS/MS) on Agilent 1200 HPLC-Chip/MS Interface (Agilent Technologies, Santa Clara, CA, USA), coupled with Agilent 6550 Accurate-Mass Q-TOF LC/MS system (Agilent Technologies, Santa Clara, CA, USA). Samples were loaded in a 75 µm × 150 mm analytical column packed with Zorbax C18 (Pore size: 300 Å, 160 nL trapping column, 5 µm particles). The sample loading volume was 1 µL and separated with 0.1% formic acid in acetonitrile using a linear flow gradient of 5–50% B for 11 min, 50–70% B for 4 min, and 70% B for 3 min. The flow rate was set to 0.4 µL/min. The drying gas flow was 11 L/min, drying gas temperature was 290 °C, fragmentor voltage was 175 V and the capillary voltage was set to 1800 V. In the positive ionization mode, spectra were acquired in an MS/MS mode with an MS scan range of 200–3000 *m*/*z* and MS/MS scan range of 50–3200 *m*/*z*. Precursor ion charge stage was set as double and above with the reference ions of 1221.9906 *m*/*z* (*z* = 1) and 299.2944 (*z* = 1). Data were extracted with an MH^+^ mass range between 50 and 3200 Da. The MS/MS data were processed with Agilent Spectrum Mill MS Proteomics Workbench software packages to provide protein and peptide identifications. Carbamidomethyl cysteine (C) was set as a fixed modification and oxidized methionine as a variable modification. The raw data was blasted against a non-redundant NCBI database of Serpentes (taxid: 8570) merged with an in-house transcript database as previously described [8]. Protein identification was validated with the following filters: protein score > 20, peptide score > 10 and score peak intensity (SPI) > 70%. Identified proteins were filtered at < 1% false discovery rate (FDR).

### 4.7. Estimation of Protein Relative Abundance

In venom fraction in-solution (FIS) profiling, the protein abundance ratio of a venom protein in its chromatographic fraction was estimated by dividing the mean spectral intensity (MSI) of its peptides by the total spectral intensity of all proteins detected in the fraction. The relative protein abundance expressed as the percentage of total venom proteins was determined by multiplying the relative spectral intensity of the protein and the chromatographic peak area under the curve (AUC) as follows:

(1) $$\mathrm{Relative}\;\mathrm{abundance}\;\mathrm{of}\;\mathrm{protein}\;y\;(\%)=\frac{\mathrm{Mean}\;\mathrm{spectral}\;\mathrm{intensity}\;\mathrm{of}\;\mathrm{protein}\;y\;\mathrm{in}\;a\;\mathrm{chromatographic}\;\mathrm{fraction}}{\mathrm{Total}\;\mathrm{spectral}\;\mathrm{intensity}\;\mathrm{of}\;\mathrm{proteins}\;\mathrm{in}\;\mathrm{the}\;\mathrm{fraction}}\times \mathrm{AUC}\;\mathrm{of}\;\mathrm{the}\;\mathrm{chromatographic}\;\mathrm{fractio}$$

### 4.8. Immunological Binding Activity Study of Antivenom

The immunological binding activity of the VINS African polyvalent antivenom (VAPAV) toward *N. senegalensis* venom was examined using an indirect enzyme-linked immunosorbent assay (ELISA) according to the method as described previously [19]. The homologous *N. haje* venom was used as a positive reference. In short, 10 ng/100 µL per well of venom antigen was pre-coated in a 96 well ELISA plate and incubated overnight at 4 °C. The plate was then flicked dry and washed three times with phosphate-buffered saline with 0.5% Tween^®^20 (PBST). Various dilutions of antivenom were added to each antigen-coated well and incubated for an hour at room temperature. After washing the plate three times with PBST, 100 µL of appropriately diluted horseradish peroxidase-conjugated antihorse-IgG (Jackson ImmunoResearch Inc., West Grove, PA, USA) in PBST (1:10,000) was added to the well and incubated for another hour at room temperature. The excess unbound antibody-enzyme conjugate was removed by rinsing three times with PBST. A hundred microliters of freshly prepared substrate solution (0.5 mg/mL o-phenylenediamine and 0.003% hydrogen peroxide in 0.1 M citrate-phosphate buffer, pH 5.0) was added to each well. The plate was left in dark for 30 min at room temperature and the reaction was terminated by adding 50 µL of 12.5% sulfuric acid. The venom-antivenom complexes were monitored at 450 nm using an ELISA reader (SUNRISE-TECAN Type Touch Screen F039300, Tecan, Männedorf, Switzerland). All experiments were performed in triplicate. The half-maximal dose (EC_50_) of the venom-antivenom binding reaction was determined from the OD values through non-linear regression analysis using Prism software version 6.0 (Graphpad Software Inc., San Diego, CA, USA)).

### 4.9. Determination of Median Lethal Dose (LD_50_) and Median Effective Dose (ED_50_)

The LD_50_ of the *N. senegalensis* venom was determined in a murine model. Various dilutions of venom in normal saline were injected intravenously into the caudal veins of ICR mice (*n* = 4 for each dose). The survival ratio was recorded after 24 h. Neutralization of the venom by the homologous antivenom was determined by preincubating a challenge dose (5 × LD_50_) of the venom with various dilutions of the antivenom at 37 °C for 30 min. The mixture was then injected intravenously into the caudal vein of mice (*n* = 4 for each dosage). Probit analysis method [82] was used to calculate the median lethal dose (LD_50_) and median effective dose (ED_50_) using the BioStat 2009 analysis software (AnalystSoft Inc., Vancouver, Canada). The potency (P) of antivenom neutralization was calculated according to Morais et al. [83]. For comparison, the neutralization potency was further normalized to normalized potency (n-P) as described previously [18,84].

### 4.10. Data Availability

The mass spectrometry proteomics data have been deposited to the ProteomeXchange Consortium3 via the iProX partner repository [85] with the dataset identifier PXD014239. 

## Figures and Tables

**Table 1:** Identification of proteins from reverse-phase HPLC fractions of *Naja senegalensis* venom through QTOF nano-ESI-LCMS/MS.

Fraction	Protein Score	Spectra/Distinct Peptide	Protein Name ^a^	Database Accession ^b^	Species	Relative Abundance ^c^ (%)
1	85.56	7/5	Short neurotoxin 1	P68417	*N. annulifera*	1.34
2	33.91	3/2	Short neurotoxin 2	P01422	*N. annulifera*	4.31
3	167.70	17/8	Long neurotoxin 1	P25674	*N. haje haje*	5.08
	100.90	10/5	Long neurotoxin 1	P01389	*N. anchietae*	5.74
	40.96	2/2	Weak toxin S4C11	P01400	*N. melanoleuca*	0.41
	36.36	5/2	Short neurotoxin 3	P01420	*N. annulifera*	0.82
4	111.54	13/6	Long neurotoxin 1	P25674	*N. haje haje*	1.69
	89.39	10/5	Long neurotoxin 1	P01389	*N. anchietae*	1.09
5	144.02	17/7	Long neurotoxin 1	P25674	*N. haje haje*	0.27
	98.09	11/5	Long neurotoxin 1	P01389	*N. anchietae*	0.22
	40.21	2/2	Weak toxin CM-2a	P25678	*N. annulifera*	0.04
6	142.81	15/7	Long neurotoxin 1	P25674	*N. haje haje*	1.19
	36.07	3/2	Weak toxin CM-2	P01415	*N. haje haje*	0.08
7	100.40	9/6	Long neurotoxin 1	P25674	*N. haje haje*	0.98
	84.19	9/5	Cytotoxin 5	P01457	*N. haje haje*	0.81
	53.20	4/4	Cytotoxin 5	P01464	*N. annulifera*	0.34
	35.48	3/2	Kunitz-type serine protease inhibitor 2	P00986	*N. nivea*	3.15
8	156.59	13/8	Cytotoxin 5	P01457	*N. haje haje*	13.56
	64.07	5/4	Cytotoxin 7	P01466	*N. annulifera*	0.32
	59.56	5/4	Cytotoxin 2	P01463	*N. nivea*	0.62
	31.29	3/2	Cytotoxin 10	P01453	*N. annulifera*	1.05
9	167.38	14/9	Cytotoxin 2	P01463	*N. nivea*	4.50
	120.82	9/6	Cytotoxin 3	P01459	*N. annulifera*	8.40
	96.59	9/5	Cytotoxin 5	P01457	*N. haje haje*	9.31
10	177.36	18/11	Cytotoxin 2	P01463	*N. nivea*	4.46
	116.09	11/7	Cytotoxin 5	P01457	*N. haje haje*	5.33
11	87.99	10/5	Cytotoxin 11	P62394	*N. haje haje*	0.83
	80.53	9/4	Cytotoxin 5	P01457	*N. haje haje*	0.90
	46.48	4/3	Cytotoxin 3	P01459	*N. annulifera*	0.78
	53.58	5/3	Cytotoxin 2	P01463	*N. nivea*	0.73
12	87.65	7/5	Cysteine-rich venom protein natrin-2	Q7ZZN8	*N. atra*	0.39
	32.94	2/2	Cysteine-rich venom protein latisemin	Q8JI38	*L. semifasciata*	0.07
	27.77	2/2	Cytotoxin-like basic protein	P62377	*N. naja*	0.04
13	30.15	2/2	nigrescease-1	B5KFV8	*C. nigrescens*	0.41
	27.97	2/2	Zinc metalloproteinase-disintegrin-like atrase-A	D5LMJ3	*N. atra*	2.56
14	87.58	9/5	Natrin-1	CL85.Contig1_NnSL	*N. naja*	2.19
	85.22	8/5	Cysteine-rich venom protein ophanin	Q7ZT98	*O. hannah*	2.63
	79.60	8/5	Cysteine-rich venom protein natrin-1	Q7T1K6	*N. atra*	2.61
	58.25	3/3	Hemorrhagic metalloproteinase-disintegrin-like kaouthiagin	P82942	*N. kaouthia*	0.42
	41.46	3/2	Metalloproteinase (Type III) 1	U3EPC7	*M. fulvius*	1.06
15	90.28	9/5	Natrin-1	CL85.Contig1_NnSL	*N. naja*	0.26
	41.20	3/3	Cysteine-rich venom protein mossambin	P0DL16	*N. mossambica*	1.07
	39.89	3/2	Metalloproteinase (Type III) 1	U3EPC7	*M. fulvius*	0.10
16	79.41	6/5	Zinc metalloproteinase-disintegrin atragin	CL626.Contig4_NsM	*N. sumatrana*	1.56
	61.97	5/4	Phosphodiesterase family member 3	U3FAB3	*M. fulvius*	2.57
	57.52	4/3	phosphodiesterase 1	CL4383.Contig2_OhM	*O. hannah*	1.04
	54.90	4/4	Cobra venom factor	Unigene31407_Nk	*N. kaouthia*	0.79
	45.85	3/3	Cytotoxin 5	P01457	*N. haje Sumatran*	0.41
	40.96	3/3	Snake venom 5’-nucleotidase	CL3600.Contig1_NsM	*N. sumatrana*	0.53
	30.17	2/2	Cytotoxin 2	P01462	*N. annulifera*	0.26
17	46.97	4/3	Metalloproteinase (Type III) 1	U3EPC7	*M. fulvius*	0.44
	36.59	3/2	Zinc metalloproteinase mocarhagin	Unigene25077_NnSL	*N. naja*	0.19
	26.93	2/2	Cobra venom factor	CL4560.Contig1_NsM	*N. sumatrana*	<0.01
	26.03	2/2	Zinc metalloproteinase-disintegrin-like atragin	D3TTC2	*N. atra*	0.03

Mass spectrometric data and peptide sequences are available in Supplemental Files S1. Abbreviations: RP-HPLC, reverse-phase high-performance liquid chromatography; nano-ESI-LCMS/MS, nano-electrospray ionization liquid chromatography-tandem mass spectrometry; *C.*, *Cryptophis*; *O*., *Ophiophagus*; *M.*, *Micrurus*; and *N.*, *Naja*. ^a^ Protein names were annotated based on matched protein homology from sequence similarity search. ^b^ Protein codes with prefix “CL” and “Unigene” were derived from the in-house RNAseq database. ^c^ Protein abundance (%) was expressed as the percentage of the total venom proteins.

**Table 2:** Overview of protein families and non-redundant proteins identified in *Naja senegalensis* venom proteome.

Protein Family/Protein Name ^a^	Database Accession ^b^	Species	Relative Abundance (%) ^c^
**3FTX**			**75.91**
SNTX			6.47
Short neurotoxin 1	P68417	*N. annulifera*	1.34
Short neurotoxin 2	P01422	*N. annulifera*	4.31
Short neurotoxin 3	P01420	*N. annulifera*	0.82
LNTX			16.26
Long neurotoxin 1	P01389	*N. anchietae*	7.06
Long neurotoxin 1	P25674	*N. haje algetic*	9.21
WTX			0.53
Weak toxin S4C11	P01400	*N. melanoleuca*	0.41
Weak toxin CM-2	P01415	*N. haje haje*	0.08
Weak toxin CM-2a	P25678	*N. annulifera*	0.04
CTX			52.64
Cytotoxin 10	P01453	*N. annulifera*	1.05
Cytotoxin 5	P01457	*N. haje haje*	30.32
Cytotoxin 3	P01459	*N. annulifera*	9.18
Cytotoxin 2	P01462	*N. annulifera*	0.26
Cytotoxin 2	P01463	*N. nivea*	10.31
Cytotoxin 5	P01464	*N. annulifera*	0.34
Cytotoxin 7	P01466	*N. annulifera*	0.32
Cytotoxin-like basic protein	P62377	*N. naja*	0.04
Cytotoxin 11	P62394	*N. haje haje*	0.83
**CRISP**			**9.23**
Natrin-1	CL85.Contig1_NnSL	*N. naja*	2.45
Cysteine-rich venom protein mossambin	P0DL16	*N. mossambica*	1.07
Cysteine-rich venom protein natrin-1	Q7T1K6	*N. atra*	2.61
Cysteine-rich venom protein ophanin	Q7ZT98	*O. hannah*	2.63
Cysteine-rich venom protein natrin-2	Q7ZZN8	*N. atra*	0.39
Cysteine-rich venom protein latisemin	Q8JI38	*L. semifasciata*	0.07
Natrin-1	CL317.Contig1_NsM	*N. sumatrana*	-
Cysteine-rich seceretory protein Bc-CRPb	F2Q6G2	*B. candidus*	-
**SVMP**			**6.78**
Nigrescease-1	B5KFV8	*C. nigrescens*	0.41
Zinc metalloproteinase-disintegrin atragin	CL626.Contig4_NsM	*N. sumatrana*	1.56
Zinc metalloproteinase-disintegrin-like atragin	D3TTC2	*N. atra*	0.03
Zinc metalloproteinase-disintegrin-like atrase-A	D5LMJ3	*N. atra*	2.56
Hemorrhagic metalloproteinase-disintegrin-like kaouthiagin	P82942	*N. kaouthia*	0.42
Metalloproteinase (Type III) 1	U3EPC7	*M. fulvius*	1.59
Zinc metalloproteinase mocarhagin	Unigene25077_NnSL	*N. naja*	0.19
**PDE**			**3.61**
Phosphodiesterase 1	CL4383.Contig2_OhM	*O. hannah*	1.04
Phosphodiesterase family member 3	U3FAB3	*M. fulvius*	2.57
Venom phosphodiesterase	A0A2D0TC04	*N. atra*	-
**KSPI**			**3.15**
Kunitz-type serine protease inhibitor 2	P00986	*N. nivea*	3.15
**CVF**			**0.79**
Cobra venom factor	CL4560.Contig1_NsM	*N. sumatrana*	<0.01
Cobra venom factor	Unigene31407_Nk	*N. kaouthia*	0.79
**5’-NUC**			**0.53**
Snake venom 5’-nucleotidase	CL3600.Contig1_NsM2	*N. sumatrana*	0.53

Abbreviations: 3FTX, three-finger toxins, CRISP, cysteine-rich secretory protein; CVF, cobra venom factor; KSPI, Kunitz-type serine protease inhibitor; PDE, phosphodiesterase; SVMP, snake venom metalloproteinase; 5′-NUC, 5′-nucleotidase. ^a^ Protein names were annotated based on matched protein homology from sequence similarity search. ^b^ Protein codes with prefix “CL” and “Unigene” were derived from the in-house RNAseq database. ^c^ Protein abundance (%) was expressed as the percentage of the total venom proteins.

**Table 3:** Lethality and antivenom neutralization of venoms from the African non-spitting cobras (genus: *Uraeus*).

*i.v*. LD_50_^a^ (µg/g)	Venom (Origin)	Antivenom	Challenge Dose	ED_50_^b^ (µL)	R ^c^ (LD_50_/_mL_)	Potency ^d^ (mg/mL)	Reference
0.39 (0.25–0.61)	*N. senegalensis* (Mali)	VAPAV	5 LD_50_	60.81 (52.77–70.08)	82.22	0.59	Present study
1.57 (1.32–1.76)	*N. annulifera* (Eswatini)	SAIMR polyvalent	3 LD_50_	52.33 (34.89–67.29)	57.33	1.20	[72]
1.57 (1.32–1.76)	*N. annulifera* (Eswatini)	Fav-Afrique	3 LD_50_	104.67 (72.46–134.57)	28.66	0.60	[72]
2.99 (2.92–3.07)	*N. annulifera* (Not specified)	Antivipmyn-Africa	3 LD_50_	304.99 (299.55–310.54)	9.84	0.39	[69]
2.35 (1.92–2.87)	*N. annulifera* (Mozambique)	VAPAV	2.5 LD_50_	125.00 (116.49–133.79)	20.00	0.65	[18]
2.35 (1.92–2.87)	*N. annulifera* (Mozambique)	PANAF	2.5 LD_50_	156.57 (127.48–190.32)	15.97	0.52	[18]
0.43 (0.35–0.52)	*N. haje* (Uganda)	SAIMR polyvalent	5 LD_50_	74.92 (73.02–77.35)	66.74	0.46	[71]
0.67 (0.61–0.73)	*N. haje* (Not specified)	Antivipmyn-Africa	3 LD_50_	73.59 (72.14–75.04)	40.77	0.37	[69]
0.84 (0.57–1.04)	*N. nivea* (Not specified)	SAIMR polyvalent	2 LD_50_	17.81 (15.58–20.35)	112.33	0.94	[70]
0.45 (0.43–0.47)	*N. nivea* (Not specified)	Antivipmyn-Africa	3 LD_50_	57.11 (56.67–57.44)	52.53	0.31	[69]

Abbreviation: *i.v.*, intravenous; LD_50_, median lethal dose; ED_50_, median effective dose; R, protective efficacy. ^a^ the dose of venom (µg/g) at which 50% of mice were dead; ^b^ the dose of antivenom (µL) at which 50% of mice survived; ^c^ the number of LD_50_ neutralized per unit volume of antivenom (mL) at which 50% of mice survived. ^d^ the amount of venom (mg) completely neutralized by per unit volume of antivenom (mL). 95% confidence intervals are indicated in parentheses.

## Data Availability

Data is contained within the article or supplementary material.

## Supplementary Materials

The following are available online at https://www.mdpi.com/2072-6651/13/1/60/s1, Table S1: Mass spectrometry analysis of in-solution tryptic digested peptides from RP-HPLC fractions of *Naja senegalensis* venom.
